# Systemic Treatment of Patients with Advanced, Unresectable Hepatocellular Carcinoma: Emergence of Therapies

**DOI:** 10.1007/s12029-018-0065-8

**Published:** 2018-02-17

**Authors:** Weijing Sun, Roniel Cabrera

**Affiliations:** 1 University of Kansas School of Medicine, Kansas City, KS USA; 2 Univesity of Kansas Cancer Center, 2330 Shawnee Mission Pkwy, Suite 210, Westwood, KS 66205 USA; 3 Division of Gastroenterology and Hepatology, University of Florida, Gainesville, FL USA

**Keywords:** Hepatocellular carcinoma, Sorafenib, Regorafenib, Nivolumab, Lenvatinib

## Abstract

To date, sorafenib, a multiple tyrosine kinase inhibitor, is the only systemic agent approved by the FDA in the first-line treatment of patients with unresectable hepatocellular carcinoma (HCC). Several other tyrosine kinase-inhibiting agents have been investigated in the first-line setting, either alone (sunitinib, brivanib, linifanib, and lenvatinib) or in combination with sorafenib (erlotinib and doxorubicin) in phase 3 trials. However, none of these studies demonstrated an improvement in survival over sorafenib. Many agents have also been tested in patients with HCC whose disease has progressed on sorafenib, but regorafenib is the only one to have demonstrated efficacy in this setting in a randomized, phase 3 trial. There were no clear survival benefits shown with everolimus, brivanib, or ramucirumab as second-line therapy. Nivolumab has also shown promising efficacy in patients with HCC who progressed on sorafenib, which was recently granted approval by the FDA, although larger confirmative trials may be considered. The treatment landscape for patients with advanced unresectable hepatocellular tumors has remained fairly static for the past 10 years, with multiple failed trials yield little change in the way these patients might be treated. However, recent findings for regorafenib, lenvatinib, and nivolumab have led to the most significant changes in the treatment paradigm in years.

## Current Treatment Paradigm for Patients with Hepatocellular Carcinoma

The incidence rate of cancers of the liver and bile duct, of which hepatocellular carcinoma (HCC) is the most common type, has increased over the past 10 years [1]. In the USA, an estimated 39,230 cases were expected to be reported in 2016, with 27,170 anticipated deaths due to cancers of the liver [2]. These statistics make cancers of the liver and bile duct the fifth leading cause of cancer-related death among patients of all ages [2]. The disease is three times more common in men than in women [3]. Faced with these statistics, multidisciplinary teams have made significant advancements in the diagnosis and treatment of patients with HCC, mainly in the areas of prevention, early diagnosis, and intervention, leading to continuous improvements in the survival rate for these patients [3].

Hepatocellular tumors represent a complex set of etiologies that can impact prognosis [3]. Multiple causes for the disease have been identified, including hepatitis B or C virus infection, alcoholic cirrhosis, non-alcoholic steatohepatitis (NASH), and aflatoxin B1 exposure [3]. Most of these conditions initiate the oncogenic transformation of liver tissue into HCC through the common pathway of liver cirrhosis although hepatitis B virus (HBV) infection may lead to HCC without the presence of cirrhosis [3]. Adding to this complexity, sex differences in the effect of HBV infection on risk for developing HCC have been identified given that the incidence of HBV-associated tumors is highest in men and post-menopausal women [4]. This effect may be due to the protective role that estrogens play through modulating inflammatory cytokines and reducing the transcription of viral RNA [4].

Importantly, the incidence of these different risk factors varies around the world, which impacts surveillance and treatment programs in different countries. For example, patients in the USA are more likely to have NASH while patients in China are more likely to have HBV infection [3]. In addition to the multiple etiologies of HCC, several different genomic pathways leading to hepatocellular tumor formation have been identified. To date, pathways related to telomere maintenance, cell cycle control, WNT-β-catenin signaling, oxidative stress, epigenetic/chromatin remodeling, AKT-mTOR-MAPK signaling, and angiogenesis have been implicated in the progression from healthy tissue to hepatocellular tumors [5–9].

Thus, patients with HCC represent a heterogenous group with a variety of etiologies and a range of presentations. Disease severity plays a critical role in selecting therapeutic regimens for this population given the range of treatments available. The tumor size and location, spread outside of the liver, and vascular invasion all must be taken into consideration when determining the optimal treatment course for any given patient.

The complexity of HCC has led to the development of an intricate staging system and set of management guidelines. The Barcelona Clinic Liver Cancer (BCLC) staging system is commonly used to guide the treatment of patients with HCC through a set of validated treatment recommendations based on the extent of a patient’s disease [10]. According to these guidelines, patients with very early (BCLC 0) or early stage (BCLC A) disease may be considered for potentially curative treatments, such as ablation, resection, or liver transplantation. Locoregional therapies, including chemoembolization and/or radioembolization, are the recommended treatments for patients with intermediate stage HCC (BCLC B) who present with more advanced disease, including the presence of large multinodular disease, Child-Pugh A or B status and Eastern Cooperative Oncology Group (ECOG) performance status (PS) 0. Once patients show massive portal invasion, extrahepatic spread, Child-Pugh A or B status, and ECOG PS 1–2 (i.e., BCLC C), the systemic therapy with tyrosine kinase inhibitor (TKI) sorafenib is the recommended treatment option. With Child-Pugh C status and ECOG PS 3 or 4, the recommended treatment options are limited to best supportive care.

Even with the benefits provided by the BCLC staging system, further refinement is needed to address its limitations and improve the treatment of patients with HCC. For example, patients may be classified as Child-Pugh A or B but have a worse prognosis due to other clinically important events, such as episodes of spontaneous bacterial peritonitis, variceal bleeding, hyponatremia, renal dysfunction, or malnutrition, which are not captured by this classification. Patients may also be classified as Child-Pugh C but satisfies the Milan criteria and therefore should be considered for liver transplant instead of best supportive care. Future efforts to refine the BCLC staging system should also incorporate molecular profiles and biomarkers into the current algorithm to more effectively guide clinical decision-making. In addition, greater knowledge of the impact of patterns of progression and development of adverse events (AEs) on treatment and survival is needed.

Sorafenib, which is recommended for patient with BCLC C status, is the first systemic therapy to demonstrate efficacy in patients with unresectable HCC [11] and has been the mainstay of care for this population. So far, no other agents have proven to be superior in first line in large phase 3 trials. Options for patients who progress on sorafenib are limited; however, recent data from the RESORCE trial indicated that regorafenib was efficacious for patients with HCC who progressed on sorafenib [12]. Nivolumab, an anti-PD-1 monoclonal antibody, has just been approved by FDA for patients with advanced HCC who were previously treated with sorafenib after showing promising efficacy and safety in a phase 1/2 trial [13].

## First-Line Systemic Treatments for Patients with Unresectable HCC

The SHARP trial was the first study to demonstrate efficacy of a systemic treatment for patients with unresectable HCC [11]. In this trial, 602 systemic treatment-naïve patients were randomized in a 1:1 ratio to either the small molecule multikinase inhibitor sorafenib at a dose of 400 mg twice daily or placebo. Treatment was continued until radiologic or symptomatic progression, the occurrence of unacceptable AEs, or death; no crossover from placebo to sorafenib was permitted.

The results showed a significantly longer median overall survival (OS) in the sorafenib group relative to the placebo group (10.7 vs. 7.9 months, HR of 0.69 [95% CI, 0.55–0.87], *P* < 0.001; Table 1) [11]. At 1-year post-randomization, 44% of patients on sorafenib were alive, compared with 33% of patients on placebo [3]. Median time to progression (TTP) was also longer in the sorafenib group (5.5 vs. 2.8 months), with 62% of sorafenib-treated patients progression-free at 4 months compared with 42% of patients in the placebo group [11]. There was no clear difference in time to symptomatic progression between the two arms [11]. Further analyses from this trial showed that the benefits of sorafenib were still present in the subgroup of patients with hepatitis C (HCV) infection [12]. In the 167 patients who tested positive for anti-HCV antibodies, median OS (14.0 vs. 7.4 months), median TTP (7.6 vs 2.8 months), and disease control rate (DCR, 44.2 vs. 29.6%) were all higher with sorafenib than in the placebo arm [12].

**Table 1:** Summary of phase 3 trials in HCC

	Investigational vs. comparator arm	OS (investigational)	OS (comparator)	TTP (investigational)	TTP (comparator)	Met primary endpoint?
First line						
SHARP [11]	Sorafenib vs. placebo	10.7	7.9	5.5	2.8	Yes
		HR 0.69 (95% CI 0.55–0.87), *P* < 0.001		HR 0.58 (95% CI 0.45–0.74), *P* < 0.001		
Asia-Pacific study [14]	Sorafenib vs. placebo	6.5	4.2	2.8	1.4	Yes
		HR 0.68 (95% CI 0.50–0.93), *P* = 0.014		HR 0.57 (0.42–0.79), *P* = 0.0005		
Sunitinib trial [15]	Sunitinib vs. sorafenib	7.9	10.2	4.1	3.8	No
		HR 1.30 (95% CI 1.13–1.50), *P* = 0.0014		HR 1.13 (95% CI 0.98–1.31), *P* = 0.3082		
BRISK-FL [16]	Brivanib vs. sorafenib	9.5	9.9	4.2	4.1	No
		HR 1.07 (95% CI 0.94–1.23), *P* = 0.3116		HR 1.01 (95% CI 0.88–1.16), *P* = 0.8532		
LIGHT [17]	Linifanib vs. sorafenib	9.1	9.8	5.4	4.0	No
		HR 1.046 (95% CI 0.896–1.221), NS		HR 0.759 (95% CI 0.643–0.895), *P* = 0.001		
SEARCH [18]	Sorafenib + erlotinib vs. sorafenib alone	9.5	8.5	3.2	4.0	No
		HR 0.929 (95% CI 0.781–1.106), *P* = 0.408		HR 1.135 (95% CI 0.944–1.366), *P* = 0.18		
CALGB 80802 [19]	Sorafenib + doxorubicin vs. sorafenib alone	9.3	10.5	NR	NR	No
		HR 1.06 (95% CI 0.8–1.4), NS		NR		
REFLECT [20]	Lenvatinib vs. sorafenib^a^	13.6	12.3	8.9	3.7	Yes
		0.92 (0.79–1.06), NR		0.63 (0.53–0.73), NR		
Second line						
BRISK-PS [21]	Brivanib vs. placebo	9.4	8.2	4.2	2.7	No
		HR 0.89 (95% CI 0.69–1.15), *P* = 0.3307		HR 0.56 (95% CI 0.42–0.76), *P* < 0.001		
REACH [22]	Ramucirumab vs. placebo	9.2	7.6	3.5	2.6	No
		HR 0.87 (95% CI 0.72–1.05), *P* = 0.14		HR 0.59 (95% CI 0.49–0.72), *P* < 0.0001		
EVOLVE-1 [23]	Everolimus vs. placebo	7.6	7.3	3.0	2.6	No
		HR 1.05 (95% CI 0.86–1.27)		HR 0.93 (95% CI 0.75–1.15), NS		
RESORCE [24]	Regorafenib vs. placebo	10.6	7.8	3.2	1.5	Yes
		HR 0.63 (95% CI 0.50–0.79), *P* < 0.0001		HR 0.44 (95% CI 0.36–0.55), *P* < 0.0001		
JET-HCC [25]	Tivantinib vs. placebo^b^	9.9	8.5	NR	NR	No
		HR 0.85 (95% CI 0.59–1.22)		NR		

Table shows the OS and TTP for the phase 3 trials reporting data in HCC. Medians are given in months

*HR* hazard ratio, *NR* not reported, *NS* not significant, *OS* overall survival, *TTP* time to progression

^a^Non-inferiority trial design

^b^Patients with c-MET high tumors only

AEs were relatively common in both groups in the SHARP trial, although most were grade 1 or 2 [11] However, diarrhea, weight loss, hand-foot skin reaction (HFSR), alopecia, anorexia, and dysphonia were observed significantly more frequently in patients treated with sorafenib than those treated with placebo. In the sorafenib arm, 26% of patients had dose reductions and 44% had dose interruptions due to AEs.

Further support for the use of sorafenib in this population was provided by an additional phase 3 trial conducted in patients from the Asia-Pacific region [14]. The results showed a significantly longer median OS of 6.5 months in sorafenib-treated patients, compared with 4.2 months in the placebo arm (HR 0.68 [95% CI 0.50–0.93], *P* = 0.014) [14]. Median TTP was also significantly longer in the sorafenib group [14]. Based on the results of the SHARP and Asia-Pacific trials, sorafenib was approved by the regulatory authorities around the world and became the standard systemic treatment for patients with advanced unresectable HCC who still have reasonable liver function (Child-Pugh A-B) [10, 26]. In addition to the SHARP and Asia-Pacific trials, GIDEON, a non-interventional study, was also conducted to further assess sorafenib effectiveness in a real-world setting [27], lending additional support to the use of sorafenib in first line.

Additional phase 3 studies have been conducted to examine other novel first-line treatments; however, to date, all have failed to meet their primary efficacy end points. Sunitinib, an inhibitor of multiple tyrosine kinases, was compared with sorafenib in 1074 patients with HCC who had no prior systemic treatment [15]. This trial was terminated after the first interim analysis when median OS was significantly shorter in the sunitinib arm relative to the sorafenib arm (7.9 vs. 10.2 months; HR 1.30 [95% CI, 1.13–1.50], *P* = 0.0014), a difference that was consistent across all pre-planned stratification groups. In addition, progression-free survival (PFS) and TTP both did not differ between arms. Grade 3 or 4 AEs occurred in 432 patients (82.1%) in the sunitinib arm and 402 patients in the sorafenib arm (74.2%). Patients on sorafenib were more likely to have dose reductions due to AEs (35.1 vs. 30.0%), but patients on sunitinib were more likely to have temporary discontinuations of treatment (76.6 vs. 58.7%).

Other novel small molecules have been compared with sorafenib in first line, including the vascular endothelial growth factor (VEGF) and fibroblast growth factor receptor inhibitor brivanib in the BRISK-FL study [16]. This study enrolled 1155 patients who were randomized (1:1) to assess the non-inferiority of brivanib compared with sorafenib. However, despite similarities in OS, the trial did not demonstrate non-inferiority of brivanib. Objective response rate (ORR) and DCR also did not show an improvement over sorafenib. The rate of discontinuation due to AEs was higher with brivanib than with sorafenib (43 vs. 33%), although the rate of dose interruptions was the same in both arms (58%).

The pattern of results was slightly altered when the VEGF and platelet-derived growth factor receptor (PDGFR) inhibitor linifanib was compared with sorafenib in 1035 patients with HCC in the LIGHT study [17]. Consistent with the other trials described above, there were no significant improvements in median OS, the primary end point, with linifanib relative to sorafenib. However, linifanib showed moderately longer TTP, PFS, and ORR (10.1 vs. 6.1%) compared with sorafenib. Patients in the linifanib arm were significantly more likely to discontinue treatment due to AEs than those in the sorafenib arm (36.3 vs. 25.4%), and dose reductions were also more common among patients on linifanib.

Investigations in first line have not been limited to single-agent regimens; the SEARCH study assessed the efficacy of sorafenib with or without erlotinib, an epidermal growth factor receptor inhibitor, for patients with HCC [18]. Median OS was 9.5 months in the combination arm and 8.5 months in the single-agent group, a difference that was not statistically significant (HR 0.929 [95% CI 0.781–1.106], *P* = 0.408). TTP also did not differ significantly between the combination and single-agent arms. In contrast to these end points, DCR was significantly higher in the single-agent sorafenib arm (52.5%) than in the group treated with sorafenib + erlotinib (43.9%), although it is important to note that treatment duration in the sorafenib arm was longer than in the combination arm (123 vs. 86 days) and therefore the longer duration of DCR could be attributed to the longer treatment with the single agent. Overall AE profiles were similar between the two treatment groups, although rash/desquamation, anorexia, diarrhea, and nose bleeding were more likely to occur in the group that received the combination of sorafenib + erlotinib.

The cytotoxic chemotherapy agent doxorubicin had been used as the systemic therapy agent for HCC without the support of a randomized study for many years before sorafenib was approved. The combination of doxorubicin with sorafenib was investigated as first-line treatment for patients with HCC in the CALGB 80802 study [19]. The study was halted when the planned interim analysis indicated that the median OS and PFS in the doxorubicin + sorafenib arm (9.3 and 3.6 months, respectively) were not significantly improved relative to sorafenib alone (10.5 and 3.2 months, respectively). Of the 38 deaths that occurred while patients were on treatment (18 in the doxorubicin arm and 20 in the sorafenib alone arm), 8 deaths in the combination arm were determined to be treatment-related, compared with only 3 in the sorafenib monotherapy arm. At the same time, AE rates were higher with doxorubicin, with 37.8% of patients reporting grade 3 or 4 hematologic AEs, compared with 8.1% of patients treated with sorafenib alone.

Most recently, the trial assessing non-inferiority of the PDGFRα, RET, and KIT inhibitor lenvatinib relative to sorafenib could potentially be considered a success, although the trial was not designed to determine if OS was significantly improved by lenvatinib [20]. In the 478 patients treated with lenvatinib, median OS was 13.6 months, compared with 12.3 months for sorafenib-treated patients (*n* = 476). Although OS was not significantly longer, some secondary endpoints favored lenvatinib over sorafenib, including PFS (7.4 vs. 3.7 months) and TTP (8.9 vs. 3.7 months). Median duration of treatment was 5.7 months in the lenvatinib arm and 3.7 months in the sorafenib arm; however, 13% of lenvatinib-treated patients discontinued treatment due to AEs compared with 9% of sorafenib-treated patients.

## Second-Line Systemic Treatments for Patients with Unresectable HCC

Options for patients who progress on sorafenib have been limited given that there were no treatments with demonstrated efficacy and safety in large phase 3 trials prior to regorafenib. In addition to the study in first line, brivanib was also investigated in patients who progressed on sorafenib in the BRISK-PS study [21]. In this study (*N* = 395), a large majority of the patients (86%) randomized to brivanib or placebo had progressed on sorafenib; the remainder were sorafenib intolerant. Median OS, the primary end point, was not significantly different between the brivanib (9.4 months) and the placebo (8.2 months) arms, even when post-study treatment was considered. However, TTP was significantly longer for patients on brivanib than on placebo (4.2 vs. 2.7 months). Discontinuation due to AEs (most often fatigue, asthenia, decreased appetite, hypertension, and vomiting) was reported in 23% of patients receiving brivanib.

Everolimus, a mammalian target of rapamycin inhibitor, was investigated in patients with HCC who progressed on or were intolerant to sorafenib in the EVOLVE-1 study [23]. Five hundred forty-six patients were randomly assigned in a 2:1 ratio to receive everolimus or placebo, the majority of whom (81.2% in the everolimus arm) had discontinued sorafenib due to disease progression. Median OS and TTP were not significantly different between patients treated with everolimus and those treated with placebo. In addition, the time to definitive deterioration of physical functioning as defined by the European Organization for the Research and Treatment of Cancer 30-item Quality of Life Questionnaire was significantly shorter for patients treated with everolimus, suggesting that this drug might impair quality of life for patients with HCC who have progressed on sorafenib. AEs leading to discontinuation occurred in 16.6% of patients treated with everolimus and 7.7% of patients treated with placebo. The most common grade 3 or 4 AEs in the everolimus group were asthenia, anemia, decreased appetite, hepatitis B infection, ascites, and thrombocytopenia.

Investigations into treatments for patients who have progressed on sorafenib have not been limited to oral agents; the REACH study assessed the intravenously administered anti-VEGFR-2 antibody ramucirumab in this population [22]. Similar to other trials in second line, 87% of the patients treated with ramucirumab had discontinued sorafenib due to progression, with the rest stopping treatment because of intolerance. Despite a 1.6-month difference in median OS between the ramucirumab and placebo groups, the difference was not statistically significant. The median PFS was significantly longer with ramucirumab (2.8 vs. 2.1 months) as was median TTP (3.5 vs. 2.6 months). Subgroup analyses from this trial suggested an increasing effect of ramucirumab with increasing baseline α-fetoprotein (AFP) values, including the pre-specified analysis comparing patients with AFP ≥ 400 ng/mL vs/ those with AFP < 400 ng/mL. Further research on the potential benefits of this agent for patients with elevated AFP is currently ongoing. The frequency of all-grade AEs was higher in the ramucirumab group than in the placebo group, with hemorrhage, hypertension, proteinuria, liver injury, and infusion-related reactions occurring more frequently in patients treated with ramucirumab than in those treated with placebo.

The JET-HCC study was designed to assess the benefits of the c-MET inhibitor tivantinib in a subset of the second-line HCC population [25]. Patients were selected for this Japanese trial because they had progressed on sorafenib and their tumors expressed high levels of c-MET protein. Unfortunately, no significant survival benefit of tivantinib was seen, with a median OS of 9.9 vs. 8.5 months with placebo. Additional follow-up may be needed to assess long-term outcomes in JET-HCC.

To date, the multikinase inhibitor regorafenib is the only agent to have demonstrated safety and significant efficacy as a second-line systemic therapy in a large, randomized phase 3 study [24]. The RESORCE trial enrolled 573 patients with documented radiological progression during treatment with sorafenib. Nearly all patients were BCLC B or C with PS 0 or 1 and Child-Pugh A liver function with the exception of 1 patient (< 1%) in the regorafenib arm who had BCLC A disease and 11 patients (2%) with Child-Pugh B liver function. All patients were required to have had treatment with sorafenib ≥ 400 mg/d for at least 20 of the 28 days prior to discontinuation. At the cutoff date for the final analysis (median follow-up of 7.0 months), median OS was 10.6 months for patients treated with regorafenib, and 7.8 months for patients treated with placebo, a statistically significant difference of 2.8 months (HR 0.63 [95% CI 0.50–0.79], *P* < 0.0001). PFS (3.1 vs. 1.5 months; HR 0.46 [95% CI 0.37–0.56], *P* < .0001) and TTP (3.2 vs. 1.5 months; HR 0.44 [95% CI 0.36–0.55], *P* < .0001) were also significantly longer with regorafenib. Patients on regorafenib also had a higher ORR (11 vs. 4%) and DCR (65 vs. 36%) relative to patients on placebo. All patients treated with regorafenib and 93% of patients on placebo experienced treatment-emergent AEs during the study. Most adverse events were grade 1 or 2. The most common grade 3 or 4 AEs (regorafenib vs. placebo) were hypertension (15 vs. 5%), HFSR (13 vs. 1%), fatigue (9 vs. 5%), and diarrhea (3 vs. 0%). Grade 5 AEs (i.e., AEs resulting in death) occurred in 13% of regorafenib patients and 20% of placebo patients.

Immuno-oncology may represent a promising new direction for the treatment of patients with unresectable HCC. The checkpoint inhibitor nivolumab was the second agent to show clinical benefits for patients with HCC in the international, non-comparative, open-label phase 1/2 CheckMate-040 study (*N* = 214 in the dose expansion phase) [13]. This trial enrolled both sorafenib-experience and -naïve patients the majority of whom had Child-Pugh A liver function (only 2% had Child-Pugh B). The ORR was 20.0% in the pooled data from patients with and without sorafenib exposure, including 3 patients (1%) with complete responses and 39 patients (18%) with partial responses. Median duration of response was 9.9 months with 6- and 9-month OS rates of 83 and 74%, respectively. Median PFS was 4.0 months, although median OS was not reached at the time of publication.

Although this trial was not randomized, the results were considered favorable enough to warrant conditional approval by the FDA for nivolumab in the treatment of patients with HCC who have progressed on sorafenib. Further research from larger, randomized trials (Table 3) will be needed to determine if these responses are durable and associated with a survival benefit. More data would also be helpful to determine the optimal line of therapy for nivolumab and other immuno-oncology agents in this population as it is currently approved for patients who have been treated with sorafenib, but the trial included some sorafenib-naïve patients as well.

## Potential Reasons for Success and Failure in HCC Trials

Understanding the potential reasons behind the success or failure of clinical trials for patients with advanced HCC can provide a useful roadmap for future developments for this population of patients with limited treatment options. Potential reasons for the failure of some of these agents include, but are not limited to, a lack of efficacy based on their mechanisms of action, the clinical heterogeneity/pathogenesis of HCC and the absence of established biomarkers capable of predicting outcomes. Also, trials in first line may be insufficiently powered to show advantages of investigational agents relative to sorafenib given that most of the agents that were evaluated were anti-angiogenic multikinase inhibitors sharing some common pathways (Table 2). Failure of these trials may have been anticipated as drugs were likely to show only marginal differences relative to sorafenib. Beyond the similarities between agents, few directly target the pathways implicated in the pathogenesis of HCC and those that do only target 1 pathway each. Importantly, there are many additional pathways, such as the insulin-like growth factor receptor, WNT/β-catenin, and hedgehog pathways [34], that are not targeted by any of these agents, including the ones that have demonstrated efficacy. A comparison of the pharmacodynamic profiles of these agents suggests that directly targeting the pathways involved in HCC may not always be necessary for extending survival in this population. Furthermore, some agents may have failed due to insufficient activity across multiple targets (i.e., erlotinib, everolimus, and ramucirumab). Lastly, the failure of everolimus and ramucirumab suggest that tyrosine kinase inhibition may be important in HCC.

**Table 2:** Targets of molecules assessed in HCC

	Pathways implicated in HCC [17]			Other targets
	EGF/EGFR	RAS/RAF/MEK/ERK	Pi3K/PTEN/AKT/mTOR	
Sorafenib [29]	–	RAF, B-RAF	–	FLT3, KIT, PDGFR, RET, VEGFR
Sunitinib [30]	–	–	–	CSF-1R, FLT3, KIT, PDGFR, RET, VEGFR
Brivanib [31]	–	–	–	FGFR, VEGFR
Linifanib [32]	–	–	–	PDGF, VEGF
Erlotinib [18]	EGFR	–	–	–
Everolimus [23]	–	–	mTOR	–
Ramucirumab [22]	–	–	–	VEGFR
Regorafenib [33]	–	RAF, B-RAF	–	FGFR, KIT, PDGFR, RET, TIE2, VEGFR
Lenvatinib [20]	–	–	–	VEGFR, FGFR, PDGFR, RET, KIT
Tivantinib [25]	–	–	–	c-MET

The table shows the known targets of the agents that have been investigated for the treatment of patients with HCC. Targets associated with pathogenesis of hepatocellular tumors are shown separately. Note that in addition to these pathways, the insulin-like growth factor receptor (IGFR), WNT/B-catenin, and hedgehog pathways as well as several inflammatory pathways have also been implicated in pathogenesis of HCC [34]

*AKT* protein kinase B, *c-MET* cellular hepatocyte growth factor receptor, *CSF* colony stimulating factor, *EGF* epidermal growth factor, *EGFR* epidermal growth factor receptor, *ERK* extracellular signal-regulated kinase, *FGFR* fibroblast growth factor receptor, *FLT* fms-like tyrosine kinase, *KIT* stem cell growth factor receptor, *MEK* mitogen-activated protein kinase, *mTOR* mammalian target of rapamycin, *Pi3K* phosphatidylinositol-4,5-bisphosphonate 3-kinase, *PDGFR* platelet-derived growth factor receptor, *PTEN* phosphatase and tensin homolog, *RAF* rapidly accelerated fibrosarcoma, *RAS* rat sarcoma, *RET* rearranged during transfection, *TIE* tyrosine kinase with immunoglobulin-like and EGF-like domains, *VEGF* vascular endothelial growth factor, *VEGFR* vascular endothelial growth factor receptor

**Table 3:** Ongoing clinical trials in HCC

Trial registration number	Description	Line of therapy
NCT02576509	Nivolumab vs. sorafenib	First
NCT02645981	Donafenib vs. sorafenib	First
NCT01737827	Capmatinib dose determination study	First
NCT01687673	Temsirolimus + sorafenib	First
NCT02524119	Ribociclib + chemoembolization	First
NCT02435433	Ramucirumab vs. placebo for patients with baseline AFP ≥ 400 ng/mL	Second
NCT02029157	Tivantinib vs. placebo for Japanese patients with high c-MET expression	Second
NCT01908426	Cabozantinib vs. placebo	Second
NCT02702401	Pembrolizumab vs. placebo	Second
NCT02329860	Apatinib vs. sorafenib	Second
NCT03062358	Pembrolizumab vs. placebo in Asian patients	Second
NCT02128958	CF-102 vs. placebo	Second
NCT02528643	Enzalutamide vs. placebo	Second
NCT02232633	BBI503	Second

The table shows selected phase 2 and 3 trials of systemic therapies for patients with hepatocellular carcinoma that are currently ongoing [28]

*AFP* α-fetoprotein

Llovet and Hernandez-Gea have suggested that sorafenib provides a clinical benefit to patients with HCC because it balances the targeting of tumor cells and their microenvironment with a manageable toxicity profile [35]. Notably, sorafenib and regorafenib are the only two agents to target RAF and B-RAF that have been investigated in HCC [29, 33]. By this rationale, regorafenib may have met its end points in second line because the partial overlap in targets with sorafenib conveyed on regorafenib the same properties of targeting the tumor and the microenvironment [29, 33].

Trial design may also play a critical role in the success or failure of clinical trials in HCC. Underlying disease characteristics, such as the extent of cirrhosis and hepatitis, that are defined in the enrollment criteria for each trial may influence the results, and future trials should be balanced with regard to these factors. In addition, further research into prognostic factors in failed trials as well as those that have succeeded should provide a better understanding of just how disease characteristics can contribute to trial outcomes. To that end, the design of the RESORCE trial may have helped facilitate the positive outcomes for regorafenib. The carefully defined criteria for prior sorafenib exposure ensured that patients were randomized to regorafenib after showing initial tolerability to sorafenib and may have improved the tolerability of regorafenib for those who enrolled. The additional requirement of randomization no more than 10 weeks after discontinuation of sorafenib should have limited progression in patients who enrolled in the trial [24].

The selection criteria for RESORCE also specified that all patients had good PS, thereby ensuring that all patients would be able to tolerate treatment [24]. Indeed, tolerability may be an important factor in the success or failure of phase 3 trials in HCC. When drugs are poorly tolerated by patients with impaired liver function, treatment-related deaths can occur, leading to worse survival in the investigational arm compared with sorafenib or placebo. This may have been the case in the study comparing sunitinib with sorafenib in first line, where approximately equal numbers of patients died due to disease progression in each arm but 18.5% of patients in the sunitinib arm and only 2.4% of patients in the sorafenib arm died due to toxicities [14].

## Conclusions and Future Directions

A look at the history of phase 3 trials for patients with HCC shows that, until the recent trials of regorafenib and nivolumab, that improving survival in this population is a high bar to pass. Numerous drugs with different mechanisms of action have attempted to demonstrate efficacy in this population and failed to meet their end points. Interestingly, many of these agents showed improvements in TTP and PFS that did not translate into prolonged OS, calling into question the clinical relevance of these end points in HCC. More data on symptomatic progression and quality of life would be helpful for determining the role of these end points in future trials.

Additional targeted and immuno-oncology agents are also currently under development and results from these trials and others have the potential to impact the future of treatment for patients with HCC. For example, ongoing research will assess the benefits of tivantinib in patients with unresectable HCC that expresses high levels of MET. Also, multiple compounds targeting FGF/FGFR, which may be related to MET signaling, are also in development. Recent results have led to the most significant changes in the treatment landscape for patients with HCC in a decade and this trend is likely to continue as new data are reported.

Future developments may also be improved by a better understanding of HCC pathogenesis, for example through genome-wide association studies. Further research into the underlying etiology of hepatocellular tumors may facilitate a more personalized approach to trial design based on biomarker expression. Additional research into how underlying liver diseases (e.g., viral infections vs. alcohol vs. NASH) may affect outcomes can also help to personalize treatment selection in HCC although it is important to note that the effects of sorafenib were observed in both patients with HBV or HCV in pooled dataset from the randomized SHARP and Asia-Pacific trials [36].

Despite previous failures, promising new developments are on the horizon for patients with HCC. Improved treatments for hepatitis may limit its contribution to HCC pathogenesis, reducing the number of cases and altering the disease characteristics of future patients with HCC such that different treatment approaches may be warranted. Immunotherapy may also play an important role in the future treatment of patients with HCC. The anti-PD-1 antibodies nivolumab and pembrolizumab as well as the anti-cytotoxic T-lymphocyte associated protein (CTLA)-4 antibody ipilimumab are all currently being investigated in different phases for patients with HCC. These agents could provide new treatment options, either alone or in combination with current TKI therapies, for this patient population.
